# Complications and management of functional single ventricle patients with Fontan circulation: From surgeon’s point of view

**DOI:** 10.3389/fcvm.2022.917059

**Published:** 2022-07-29

**Authors:** Jianrui Ma, Jimei Chen, Tong Tan, Xiaobing Liu, Rong Liufu, Hailong Qiu, Shuai Zhang, Shusheng Wen, Jian Zhuang, Haiyun Yuan

**Affiliations:** ^1^Department of Cardiovascular Surgery, Guangdong Cardiovascular Institute, Guangdong Provincial People’s Hospital, Guangdong Academy of Medical Sciences, Guangzhou, China; ^2^Shantou University Medical College, Shantou, China

**Keywords:** single ventricle, Fontan, complication, treatment, congenital heart disease

## Abstract

Fontan surgery by step-wise completing the isolation of originally mixed pulmonary and systemic circulation provides an operative approach for functional single-ventricle patients not amenable to biventricular repair and allows their survival into adulthood. In the absence of a subpulmonic pumping chamber, however, the unphysiological Fontan circulation consequently results in diminished cardiac output and elevated central venous pressure, in which multiple short-term or long-term complications may develop. Current understanding of the Fontan-associated complications, particularly toward etiology and pathophysiology, is extremely incomplete. What’s more, ongoing efforts have been made to manage these complications to weaken the Fontan-associated adverse impact and improve the life quality, but strategies are ill-defined. Herein, this review summarizes recent studies on cardiac and non-cardiac complications associated with Fontan circulation, focusing on significance or severity, etiology, pathophysiology, prevalence, risk factors, surveillance, or diagnosis. From the perspective of surgeons, we also discuss the management of the Fontan circulation based on current evidence, including post-operative administration of antithrombotic agents, ablation, pacemaker implantation, mechanical circulatory support, and final orthotopic heart transplantation, etc., to standardize diagnosis and treatment in the future.

## Introduction

Functional single ventricle (FSV) is a heterogeneous spectrum of congenital heart disease (CHD), encompassing double inlet atrioventricular connection, absence of one atrioventricular connection, unbalanced common atrioventricular canal defect, single ventricular heterotaxia syndrome, and hypoplastic left heart syndrome (HLHS). As the most complex CHD, it is uniformly fatal without intervention.

Fontan surgery that separates pulmonary and systemic circulation by the creation of venous-to-pulmonary connection for passive delivery of deoxygenated blood into the lungs and utilization of the sole FSV to maintain the systemic circulation, provides palliative strategies for FSV patients. It was first described by Fontan and Baudet in 1971 to innovatively repair tricuspid atresia (1). Since then, the Fontan surgery has experienced multiple modifications. The atrioventricular and atriopulmonary (AP) connections were initially attempted to achieve Fontan circulation. However, both of them have been discarded due to energy loss, flow disturbance, and arrhythmic burden associated with significant morbidity and mortality. An innovative concept in terms of total cavopulmonary connection (TCPC) was thereby proposed by generating the connection, including the atrial lateral tunnel (LT) and later intracardiac or extracardiac conduit (EC) for flow hemodynamic optimization, which is the most widely employed at present (2). Further evolution of Fontan surgery was the generation of fenestration, allowing a tailored right-to-left shunt to retain cardiac output in patients with high risk (3). This modification that enables in part offloading the systemic venous pressure, as well as increasing the ventricular preload has been routinely adopted in some institutions as a consequence of improved clinical benefits.

Despite enormous progress, the distinct Fontan circulation in the absence of a sub-pulmonic pumping chamber will lead to diminished cardiac output, elevated central venous pressure, and non-pulsatile pulmonary blood flow, thereby inevitably a cascade of short-term or long-term complications. Considerable efforts have been made to investigate the possible complications and explore possible therapeutic interventions, aiming to minimize the adverse effect, standardize the post-operative management, and improve the life quality of Fontan patients.

Therefore, in this review, we discuss the latest research on cardiac and non-cardiac complications associated with Fontan circulation, involving significance or severity, etiology, pathophysiology, prevalence, risk factors, surveillance or diagnosis, and general treatment. What’s more, current management strategies including post-operative anticoagulation agent administration, ablation, pacing, mechanical circulatory support (MCS), and heart transplantation are also introduced from the surgeon’s point of view.

## Complications

### Atrioventricular valve regurgitation

Atrioventricular valve regurgitation (AVVR) is a frequent cardiac complication in Fontan circulation, which adversely impacts long-term outcomes in terms of increasing the risk of heart transplantation or death. Although the underlying pathophysiology remains incompletely clear, AVVR is considered to be attributed to a structural aberration of valve apparatus, as well as functional ventricular alteration. Structural abnormalities dominantly lead to AVVR and may take place at any part of the valve apparatus, such as abnormal commissures and clefts, shortened or elongated chordae, and dysplastic leaflets. As for functional etiologies, Fontan surgery exposes patients to a chronic volume overload resulting from aortic valve regurgitation or aortopulmonary collaterals, so that AVVR may evolve over time. Alteration in hemodynamics at different stages can also result in dysfunction of valve apparatus. It was verified that Fontan patients with common atrioventricular valves inherently suffered from sustained attrition of valve function (4).

Imaging modalities that can accurately assess valve anatomy and function encompass transthoracic echocardiography (TTE), transesophageal echocardiography (TEE), 3-dimensional echocardiography, computed tomography (CT), and cardiac magnetic resonance (CMR). Multiple modality imaging is recommended before next-step decision-making as each type of imaging modality has a unique advantage, as well as constraints. However, management strategies for AVVR have not been well-established. Drug treatment including diuretic therapy and afterload reduction can be attempted, but more evidence concerning the effect of these medications is required. Surgical intervention should be considered if AVVR seriously impacts patients’ life quality even after pharmacotherapy. AVVR historically used to be regarded as a contraindication for Fontan surgery. Over the years, concomitant repair or replacement of AVV during Fontan surgery has gradually been performed nowadays. The strategies of surgical intervention in terms of regurgitation severity stratification, as well as optimal timing for AVVR intervention, are not well-established. AVV repair is recommended in those with FSV and atrioventricular septal defects if AVVR exceeds a moderate degree in some centers.

### Arrhythmia

Arrhythmia is a common and troublesome event after Fontan surgery, including atrial tachyarrhythmia, supraventricular tachyarrhythmia, and bradyarrhythmia. In particular, atrial tachyarrhythmias are poorly tolerated in FSV patients with Fontan circulation, potentially leading to a deterioration of AVVR, heart failure, thrombogenesis, and even sudden death. Suture lines, atrial scar, chronic atrial dilation, increased atrial pressure, and long-term cyanosis are associated with the development of atrial tachycardias, while bradyarrhythmia is considered to be primarily attributed to the direct sinus node injury or reduction of its blood supply. The mechanism of arrhythmia after Fontan surgery is diverse, covering the atrioventricular reentry *via* a second atrioventricular node or an accessory pathway, atrioventricular nodal, focal atrial tachycardia, and reentrant circuits. There was also a distinctly deranged autonomic nervous control in the heart in terms of reduced baroreflex sensitivity and heart rate variability after Fontan completion. FSV individuals with prominent right atrial dilation presented distinct suppression of the sympathetic system in contrast to the parasympathetic system (5).

Given the adverse impact on the outcome as well as the high prevalence of arrhythmia, regular monitoring and ideal management are of paramount. Electrocardiograms are recommended by 2019 American Heart Association guidelines to perform every 6–12 months in such populations (6). In addition, Holter monitors are advised to be performed in children every 2–3 years and adolescents and adults every 1–2 years (7). As for those presenting with worsening or new-onset arrhythmia, prompt comprehensive assessment by detailed history collection, thorough examination, 12-lead electrocardiogram, TEE or TTE, and cardiac catheterization is required to rule out potential responsible factors like intracardiac shunts, regurgitant or obstructive lesions, myocardial ischemia, and ventricular failure. Early referral for multidisciplinary treatment is crucial once arrhythmia is confirmed. Acute arrhythmia management includes stabilizing the hemodynamics, cardioversion, pacing, or medication initiation. While chronic management strategies involve rhythm control for tachyarrhythmias and pacemaker placement for bradycardia or sinus node dysfunction, concomitant surgical intervention of atrial arrhythmia can be performed during the Fontan conversion with Class I recommendations (8). Fontan conversion and catheter ablation are superior in the prevention of recurrence of atrial tachyarrhythmia in contrast to conservative therapy like intake of antiarrhythmic drugs.

### Ventricular dysfunction

Systolic or diastolic ventricular dysfunction is a common complication that deteriorates long-term mortality and morbidity. Systolic and diastolic ventricular dysfunctions can occur simultaneously due to the strong interdependence in FSV physiology. The etiology is multifactorial, covering chronic hypoxia, pressure and volume overloading, and multiple surgeries. Although the mechanism remains poorly elucidated, myocardial fibrosis remodeling responsive to chronic stress is thought to play an important role in the development of FSV dysfunction. Growing evidence has suggested that the myocardial fibrosis remodeling leads to an impairment of ventricular contractility and compliance, therefore compromising both the systolic and diastolic FSV. However, Nakano et al. compared the histologic fibrosis and fibrotic-associated gene expression among the diverse type of FSV patients. The results showed that neither fibrotic expression profile nor histologic fibrosis was a prominent characteristic in young FSV patients with RV dysfunction, raising doubts regarding the main contribution of fibrotic remodeling in RV dysfunction in such population (9). The role of increased ventricular dyssynchrony inherently with Fontan patients that is conventionally measured by speckle-tracking echocardiography is increasingly recognized. It was reported that more than 15% of adult Fontan subjects suffered from classic pattern dyssynchrony (10). The ventricular dyssynchrony also varied among Fontan types, worse in AP Fontan in contrast to EC Fontan, probably due to the chronic severe reduction of ventricular preload in the AP Fontan subjects (11). Both increased ventricular electrical and mechanical dyssynchrony was in part associated with myocardial deformation, myocardial remodeling, and further ventricular systolic or diastolic dysfunction (10, 12). Novel imaging modalities like CMR with feature tracking that enables reliable quantification of ventricular mechanics would be helpful to discover the interplay between ventricular dyssynchrony and ventricular dysfunction in Fontan patients.

The dependable quantified assessment of ventricular function in Fontan patients is full of challenges owing to unique and intricate cardiac morphology, anatomy, and physiology. N-terminal pro-brain natriuretic peptide (NT-proBNP) and Brain Natriuretic Peptide (BNP) are clinically common biomarkers to identify cardiac dysfunction. It is also applicable to FSV physiology and further reflects the extent of hemodynamic overloading, as well as the long-term outcome. However, BNP and NT-proBNP levels have been elevated before Fontan completion. And there was no significant difference in the BNP levels between asymptomatic ventricular dysfunction FSV patients and healthy controls. Therefore, the BNP and NT-proBNP are more appropriate for detecting the progression of heart failure by sequential measurement in FSV physiology (13). Conventionally, echocardiographic indices like ejection fraction (EF) and shortening fraction based on 2D or M-model are commonly used for the evaluation of LV function, which is also applicable to LV-dominant Fontan patients to assess the ventricular function and size. Given the correlation of reduced torsion with systolic ventricular dysfunction, speckle-tracking echocardiography that can measure myocardial strain and torsion, may also provide a supplementary tool in assisting the evaluation of ventricular function in Fontan circulation. However, the normal values to assess ventricular function for FSV patients under echocardiographic examination are not well defined. Interventional modalities and advanced imaging are thereby recommended for better quantification if ventricular failure is suspected. Albeit with invasive and trauma concerns, cardiac catheterization by measuring the end-diastolic pressures is considered a standard in quantifying the diastolic function in FSV physiology. CMR is a reference standard modality in quantifying the ventricular mass, volume, and EF owing to the capacity of accurately assessing the myocardial structure, geometry, and function. In addition, compared with catheterization, it is superior in the evaluation of cardiac output, as well as the relative flow, that is susceptible to a diverse origin of pulmonary flow in FSV patients. Considering that EF is partially dependent on cardiac chamber loading conditions and insensitive to early identification of heart failure, strain, independent of ventricular morphology and geometry, may be a superior parameter to assess the myocardial deformation and ventricular function in FSV physiology. It enables early identification of systolic ventricular function impairment in asymptomatic Fontan failure where cardiac index and EF are still preserved.

The diuretics regimen to reduce the afterload is efficient to relieve symptoms in this setting. But there is debate over whether beta-blockers or renin-angiotensin-aldosterone inhibitors benefit the treatment of ventricular dysfunction in Fontan patients.

### Fontan-associated liver disease

Fontan-associated liver disease (FALD), an array of congestive hepatopathy consisting of liver fibrosis, cirrhosis, and even carcinoma, is a ubiquitous non-cardiac complication encountered in FSV patients under unique Fontan hemodynamics. The natural history has been well described, mainly encompassing three main stages, as summarized in Table 1 (14). Although the mechanism of FALD development is intricate and remains to be further elucidated, the hemodynamic alteration related to Fontan surgery in terms of elevated central venous pressure and diminished cardiac output is heavily responsible. To maintain the liver sufficient perfusion, an intricate hepatic arterial buffer response would be triggered to promote great arterialization. Herein, more proportion of liver blood supply is originated from the hepatic artery, which may lead to hepatic morphologic and functional alteration like arterialized nodules. Additionally, the elevated central venous pressure secondary to the absence of a pumping chamber is transmitted to the sinusoid, resulting in sinusoidal hyperfiltration, dilation, and eventually perisinusoidal edema. In this context, the increased sinusoidal shear stress would enhance the hepatic stellate cell activation, contributing to liver fibrosis (15). It was also clear that chronic liver congestion enhanced hepato-fibrosis and HCC growth mediated by upregulation of SphK1/S1P/S1PR pathways in lipopolysaccharide-induced capillarized liver sinusoidal endothelial cells (16).

**TABLE 1:** Progression of Fontan-associated liver disease.

Stage	Period	Clinical manifestation	Analytics	Histology
**Stage I: Sinusoidal congestion without fibrosis**	Before FS or less than 10 years after FS	Asymptomatic or painful hepatomegaly Weight loss Anorexia Lethargy Hepatojugular reflux	Mild elevated AKP Mild elevated GGT Mild elevated indirect Bilirubin Reduced LDL-C, HDL-C, and total cholesterol	Sinusoidal dilatation Hepatocellular necrosis Mild perisinusoidal fibrosis
**Stage II: Fibrosis without portal hypertension**	About 10–15 years after FS	Asymptomatic or painful hepatomegaly Weight loss Anorexia Lethargy Hepatojugular reflux	Elevated AKP, GGT, indirect Bilirubin Mild elevated AST, ALT, LDH Reduced LDL-C, HDL-C, and total cholesterol	Sinusoidal dilatation Hepatocellular necrosis Moderate perisinusoidal fibrosis Regenerative nodules
**Stage III: Terminal fibrosis with or without portal hypertension**	Over 20 years after FS	Jaundice Ascites Gastroesophageal varices Hepatic encephalopathy HCC	Hyperbilirubinemia Thrombocytopenia Reduced albumin and prothrombin Prolonged INR	Central-central bridging Fibrosis Regenerative nodules

FS, Fontan surgery; AKP, alkaline phosphatase; GGT, gamma-glutamyl transpeptidase; LDL-C, low-density lipoprotein cholesterol; HDL-C, high-density lipoprotein cholesterol; AST, aspartate aminotransferase; ALT, alanine transaminase; HCC, hepatic cell carcinoma; INR, international normalized ratio.

Regular and comprehensive surveillance is crucial to identify the development of FALD. It is recommended that Fontan patients with HCC are supposed to undergo cancer screening every 6 months. Liver biopsy represents the gold standard in identifying and assessing hepato-fibrosis in FALD. However, the biopsy-associated bleeding complication on account of elevated systemic venous pressure and the frequent use of anticoagulation drugs may limit its universal surveillance in FALD. Non-invasive surveillance tools like serology and imaging modalities may be complementary. Liver enzymes are frequently normal until the late FALD, failing to early identify FALD and assess its severity. Model for end-stage liver disease (MELD), Pediatric End-stage Liver Disease (PELD), and aspartate-to-platelet ratio index (APRI) have been verified as useful serum markers as they are correlated with liver stiffness and fibrosis. However, these tools relied on serum INR derangements, thereby not applicable in those Fontan patients with warfarin therapy. Alternatively, the MELD XI score that excludes INR and uses serum creatinine and serum bilirubin may also monitor and assess FALD in such population. Imaging modalities are capable of non-invasively providing detailed, complementary, and accurate assessments of liver structure, morphology, and hemodynamics in Fontan patients. Although it is relatively insensitive in identifying the fibrotic alteration, abdominal ultrasonography (US) is an important evaluation imaging modality as a consequence of providing gross structural features and hemodynamic information. Heterogeneous surface nodularity or hepatic echotexture was a prevalent finding, which was correlated with time after the Fontan surgery (17). Besides, augmented liver volumes associated with congestive hepatopathy in the early period and decreased liver volumes associated with liver cirrhosis in the late period could also be identified under the abdominal US. When transhepatic resistance or central venous pressure increases, imaging features from Doppler tracings included alterations in mesenteric and celiac flow resistances, hepatic artery enlargement due to the arterialization, and the absence or presence of anterograde portal flow (7). In contrast, both CT and magnetic resonance imaging (MRI) are better able to detect a liver injury or scarring, architecture, and mass in detail. Predominantly peripheral, hypervascular, and hyperechoic nodularity and liver lobulated appearance on MRI are universal morphologic characteristics in Fontan patients. These tools also yield information associated with portal hypertension: altered liver periphery enhancement and reticular parenchyma heterogeneous enhancement in the portal venous phase, venous collaterals, ascites, and hepatic vein dilation with contrast reflux. Elastography also merits the evaluation of the FALD because of the advantage of non-invasively measuring liver stiffness, in which transient elastography, shear wave elastography, and MRI elastography are currently major employed modalities. FSV patients with a longer time from the Fontan surgery have been demonstrated with increased liver stiffness determined by the elastography, indicating more severe liver fibrosis (18). However, the clinical utility is hampered by the inability to determine the contribution of hepatic congestion and fibrosis to the increased liver stiffness. New cutoffs of liver stiffness should be defined and confirmed to allow the diagnosis of FALD with better specificity.

Despite histological findings and imaging results, decision-making is hard because of the failure to accurately assess residual liver function. The prevention and treatment of FALD are dependent on the Fontan circulation optimization by medical or surgical interventions to reduce CVP or increase cardiac output. Heart-only transplantation is adequate and suitable for those FALD patients with only mild portal hypertension, normal hepatic vessel anatomy, preserved liver synthetic function, liver volume more than 800 ml, but no HCC. If there is advanced liver cirrhosis evidence in terms of clinical manifestations, imaging results, or biopsy findings, and if there is certain decompensation evidence, combined heart-liver transplantation can be taken into consideration as an alternative (19).

### Renal dysfunction

Normally, the blood supply of kidneys accounts for 20–25% of heart output at rest. Herein, the Fontan hemodynamic alteration may damage the kidneys, leading to renal dysfunction including acute kidney injury (AKI) and chronic kidney disease (CKD). Cardiopulmonary bypass, use of nephrotoxic medications, Fontan unique hemodynamic in terms of low cardiac output and elevated venous pressure, and hypoxemia may play a dominant role in the development of renal dysfunction. Fontan-related renal dysfunction seemed to be tolerable in the early post-operative period as similar 10-year survival rates were observed between Fontan patients with and without Fontan renal dysfunction (20). However, it could lead to approximately 10% of mortality in the 20-year follow-up (21). Clinical identification and assessment of CKD highly rely on eGFR, although it might fail to accurately identify those with mild renal dysfunction through direct GFR measurement. Some endogenous markers such as Cystatin C and creatinine have been extensively applied for GFR estimation by related equations. Given that FSV patients have poor nutritional status and muscle mass, Cystatin C may be a more reliable and precise indicator than serum creatinine in estimating renal function. In addition, cystatin C-calculated eGFR appeared to be a better indicator than creatinine-calculated eGFR to predict all-cause mortality (22). Indexed IVC diameter through echocardiography was shown as a novel and non-invasive marker in identifying those at risk for Fontan nephropathy as it was strongly correlated with microalbumin–creatinine ratio (23). Kidney injury molecule-1 and *N*-acetyl glucosaminidase could also be considered to be routinely detected as they could not only effectively identify Fontan-associated tubular injury but also predict adverse outcomes in terms of non-elective cardiovascular death or hospitalization (24). Although some medicine like ACEI has been attempted to treat Fontan-associated renal dysfunction and showed plausibly satisfactory results, there are no effective tactics to prevent and treat this troublesome complication.

### Thromboembolic events

Thromboembolic events (TE) are a significant cause of morbidity and mortality in FSV patients with Fontan circulation. Although the development and mechanism of TE are incompletely defined, three factors of Virchow’s triad are involved: endothelial dysfunction, abnormal hemodynamics, and hypercoagulability. Specifically, endothelial dysfunction refers to the loss of antithrombotic factors, including Protein C, S, and antithrombin, and vasodilators like nitric oxide, as well as the increased prothrombotic products and vasoconstrictors, eventually resulting in a pro-thrombotic condition. The hemodynamics also alters, manifesting as often turbulent and non-pulsatile flow, which may predispose Fontan patients to develop TE. The reduction of antithrombin III, fibrinogen, protein C, plasminogen, factor II, V, VII, IX, and X were detected in both the pre-Glenn and post-Fontan patients, suggesting the hypercoagulable state due to an imbalance between coagulation and anticoagulation system occurred prior to Fontan surgery and maintained throughout the entire post-Fontan period (25, 26). Liver function impairment that reduces the synthesis of protein C and S, and protein-losing enteropathy (PLE) that impairs the clotting cascade due to a substantial loss of serum proteins into the digestive tract, might also exacerbate the hypercoagulable state of Fontan patients.

Given the significant association with mortality, the early diagnosis of TE is of paramount. D-dimer detection might be useful in identifying intracardiac thrombus in Fontan circulation. The proper cutoff value to rule out thrombus within the cardiac chamber was 1.8 μg/mL, with 85% sensitivity and 92% specificity (27). Imaging tools like TTE can be employed to identify intracardiac thrombus but are easily hindered by chest tubes, dressings, and edema after the surgery. In contrast, TEE is extensively employed in detecting thrombi within the ligated pulmonary artery, systemic ventricle, and systemic venous pathway as a consequence of providing a more accurate and clearer image than TTE. MRI and MRI angiography might also afford an option to early recognize pulmonary embolism and assess the Fontan pathway patency; However, the application is limited because of MRI artifacts resulting from pacemaker leads or coils and the examining time-sensitivity. Alternatively, computed tomographic angiography (CTA) is a proper approach to detect Fontan-related pulmonary embolism on account of ideal sensitivity and specificity. There would be suboptimal opacification within the Fontan pathway or pulmonary arteries when examined by CTA due to anatomic and hemodynamic changes associated with Fontan surgery. Thus, acquisition of CT imaging in the early and delayed contrast enhancement phases followed by contrast agent injection *via* both an upper and lower extremity is recommended to improve imaging quality.

The pharmacotherapy medication to prevent and treat thromboembolic complications predominantly relies on the control of atrial arrhythmias, as well as thromboprophylaxis. It has been recommended with 2C Class by the American College of Chest Physician Evidence-Based Clinical Practice Guidelines for Antithrombotic Therapy and the Prevention of Thrombosis to routinely post-operative use of unfractionated heparin (UFH) in those neonates and children with either bilateral cavopulmonary shunt or bidirectional Glenn operation. Despite the recommendations, thromboprophylaxis strategies vary among centers, encompassing the single-use of aspirin, warfarin, UFH, and enoxaparin, or combined use of aspirin and low-molecular-weight heparin (LMWH), aspirin and warfarin, UFH and aspirin, and UFH and LMWH.

### Protein-losing enteropathy

Protein-losing enteropathy is an unusual but devastating complication with a remitting and relapse course, characterized by massive loss of protein into the digestive tract leading to low total protein and serum albumin levels, hypercoagulability, and immunodeficiency on account of enteric loss of lymphocytes and immunoglobulins. Patients may manifest abdominal cramps, chronic diarrhea, malnutrition, peripheral edema, persistent pleural effusions, ascites, and decreased bone mineral density. The underlying pathophysiology and etiology appear to be multiple. First, elevated systemic venous pressure resulting from pulmonary artery branch stenosis, Fontan pathway obstruction, and elevated atrial pressures associated with arrhythmias, AV-valve regurgitation, and ventricular diastolic dysfunction would predispose Fontan patients to generate PLE (28). Secondly, the diminished cardiac output might compromise intestinal perfusion and raise mesenteric vascular resistance, thereby jeopardizing gut mucosal integrity (28). Additionally, a recent study showed that there was an abnormal formation of hepatoduodenal lymphatic connections leading to liver lymph leakage into the digestive tract (29). Lymphatic congestion frequently encountered in Fontan patients would further aggravate the lymphatic leak from the high-pressure lymphatic vessel into the low-pressure tract. Other factors like bowel inflammation and low pulmonary vascular compliance were also involved in the development of PLE (30, 31). The substantial enteric protein loss leads to systemic edema, including the intestinal wall, so that nutrition absorption will be impaired and protein loss is exacerbated, eventually resulting in a vicious cycle.

Elevation of α-1 antitrypsin clearance in 24-h collected stool samples remains the gold standard test in PLE diagnosis. Because of the difficulty in collecting sufficient stool samples, it is also agreed with alternative criteria in terms of clinical edema, hypoalbuminemia together with an elevated α-1 antitrypsin based on a single stool specimen after excluding other causes. Technetium-99 m labeled human serum albumin scintigraphy has also been adopted to image and diagnose the PLE since its first attempt in 1986 (32). It provides several advantages, including low cost, non-invasiveness, simplicity, and the ability to localize the site of enteric protein loss. Previous studies have demonstrated its higher sensitivity and negative predictive value in diagnosing PLE than the fecal α-1 antitrypsin clearance (33). However, the specificity is suboptimal thereby hampers the application of this imaging tool. Once the PLE is diagnosed, it will be essential to detect reversible abnormal hemodynamics through imaging tools, including TTE, TEE, cardiac catheterization, and MRI. Hemodynamic abnormalities like severe aorta coarctation and AVVR are highly recommended to be corrected.

Although the management and treatment in PLE patients remain a challenge, the outcome has been improved with increased survival of 88 and 72% at 5 and 10 years, respectively, with the recent advancement in treatment strategies (34). To date, the treatment of PLE including medication, diet modification, interventional, and surgical therapies focus on relieving symptoms, minimizing the side effect of reagents, and improving life quality. The medication therapy is various, consisting of stabilization of intestinal cell membrane with heparin, targeted pulmonary vasodilators, albumin infusion, volume overload reduction with diuretics, and anti-inflammation with steroids. Diet modification can also benefit PLE patients *via* a combination of low sodium, high medium-chain triglycerides, low fat less than 25% calories, and high protein over 2 g/kg/d (35). Interventional therapy attempts to reduce volume overload *via* aorto-pulmonary collateral embolization and removal of pathway obstruction *via* fenestration creation or stent implantation. While surgical therapy involves atrial pacing implementation, fenestration creation, and paralyzed diaphragm plication. Heart transplantation may represent a curative and ultimate strategy for PLE as it eliminates the diminished cardiac output and elevated systemic venous pressure.

### Plastic bronchitis

Similar to PLE, plastic bronchitis (PB) is a sparse but potentially fatal disorder in Fontan patients, characterized by the generation and expectoration of cohesive and obstructive bronchial casts. But PB can occur more acutely and perilously in contrast to PLE. The cast is composed of dominating mucinous components but rare chylous and inflammatory components, with sizes ranging from a small segmental bronchus to the whole bronchial tree. In this context, patients may have wheezing, shortness of breath, coughing, chest pain, and even fatal asphyxia. Although the pathogenesis of PB in Fontan patients remains poorly understood, it may share some mechanisms with PLE. Inherent hemodynamic changes in Fontan patients in terms of elevated systemic venous pressure, diminished cardiac output, and elevated pulmonary artery pressure are responsible for the injury of the alveolar-capillary barrier and mucosal integrity, which consequently leads to cast formation *via* leakage of cellular and proteinaceous components. Respiratory tract inflammatory response secondary to the inherently abnormal inflammatory response or simultaneous infection leads to the fibrin cross-linking within the lymph and therefore the formation of the solid and rubbery cast. Surgical injury of the thoracic duct by accident places patients at risk of PB development owing to abnormalities in lymphatic fluid drainage and excessive generation.

There would be possible opacification of the involved lung field owing to full or partial atelectasis on chest X-ray examination, which is not sensitive and specific enough. The current diagnosis of PB is based on the evident bronchial casts by expectoration or bronchoscopy removal. Lymphangiography capable of discovering aberrant lymphatic vessels, such as thoracic duct dilation, lymphatic collateral formation, and lymphangiectasia, can be a complementary tool in confirming the PB diagnosis. In general, the strategy of PB management differs from the clinical status. The acute attack of PB should maintain the airway unimpeded immediately by promoting cast expectoration, as well as invasive removal of the cast. While the primary focus of chronic PB is to maintain respiratory tract clear and identify the underlying reason for a cast. The pharmacological therapy for PB now is highly anecdotal, covering the bronchodilators, antibiotics, corticosteroids, mucolytics, aerosolized heparin, tissue plasminogen activator, and urokinase in a myriad of integration. Given the role of lymphatic circulation in the development of PB, percutaneous embolization of abnormal lymphatic fistulae that enabled the prevention of lymphatic leakage showed satisfying outcomes and thereby might become a promising interventional treatment (36). The combination of anti-inflammatory therapy, relief of respiratory tract obstruction, and hemodynamic improvement including relief of residual obstruction, augmentation of cardiac output, and reduction of lymph production are the predominantly acceptable and effective treatments at present.

## Management

Cardiac and non-cardiac complications related to Fontan surgery are summarized in Table 2. Given the adverse impact of Fontan-associated complications on Fontan outcomes, several clinical strategies have been adopted to minimize and manage these troublesome complications in Fontan circulation or failure (Table 3).

**TABLE 2:** Summary of cardiac and non-cardiac complications associated with Fontan circulation.

Complications		Incidence rate	Risk factors	Examination
Cardiac	AVVR	10–39.3%	Extracardiac conduit, Right ventricle morphology, Presence of collaterals, Fontan fenestration, Pre-operative impaired ventricular function	TTE, TEE, 3D echocardiography, CT, CMR
	Arrhythmia	60%	Older age at Fontan surgery, Paced rhythm, Atriopulmonary connection, Moderate or greater AVVR, Lower functional status	Holter, TEE, TTE, Cardiac catheterization
	Ventricular dysfunction	50%	Increased volume loading, Right ventricle morphology, Prolonged cumulative bypass time, AVVR	BNP, pro-BNP, TEE, TTE, Speckle-tracking echocardiography, Cardiac catheterization, CMR
Non-cardiac	FALD	56.6% at 20-year follow-up 98.9% at 30-year follow-up	Longer duration of Fontan exposure, Older age, Higher Fontan pressures, Alcohol abuse, Hepatitis B or C, Use of hepatoxic drugs	Liver biopsy, Serology, US, CT, MRI, Elastography
	Renal dysfunction	8%	Elevated post-bypass Fontan pressure, Intraoperative arrhythmias, Elevated pre-operative pulmonary artery pressure, Pre-operative AVVR, Mean renal perfusion, Asplenia	GFR measurement
	Thromboembolic events	30%	Older age, Increased liver stiffness, Lower post-operative FiO2 24 h, Prolonged adoption of central venous lines, Lower unconjugated bilirubin before surgery, Pulmonary artery distortion, Pulmonary atresia with intact ventricular septum	D-dimer detection, TTE, TEE, CT, CTA, MR, MRA
	PLE	3.7–11.3%	Post-operative pleural effusions, Older age at Fontan surgery, Right ventricle morphology	α-1 antitrypsin clearance, Technetium-99 m labeled albumin scintigraphy, TTE, TEE, Cardiac catheterization, MRI
	PB	4%	Chylothorax, Long duration of chest tube drainage, Major cardiac operations, Right ventricle morphology, Ascites	Chest X-ray examination, Bronchoscopy, Lymphangiography

AVVR, atrioventricular valve regurgitation; FALD, Fontan-associated liver disease; PLE, protein-losing enteropathy; PB, plastic bronchitis; TCPC, total cavopulmonary connection; FiO2, fraction of inspiration O2; TTE, transthoracic echocardiography; TEE, transesophageal echocardiography; CT, computed tomography; CMR, cardiac magnetic resonance; CTA, computed tomographic angiography; BNP, brain natriuretic peptide; NT-proBNP, N-terminal pro-brain natriuretic peptide; US, ultrasound; MRI, magnetic resonance imaging; GFR, glomerular filtration rate; MRA, magnetic resonance angiography.

**TABLE 3:** Benefits and concerns of the management associated with Fontan circulation.

Management	Benefits	Concerns
Thromboprophylaxis	Lower TE rate Lower risks of hospitalization and death	Increased risks of bleeding Bone mineral density reduction Cerebrovascular injuries
Ablation	Arrhythmic burden elimination Less discomfort Lower mortality Shorter hospital stay	Low success rate High recurrence rate Complete atrioventricular block Residual inducible arrhythmias
Pacing	Hemodynamic improvement Symptom relief Antiarrhythmic effect improvement	Limited venous access Necessity of sternotomy or thoracotomy Disappointing outcome
ECMO	Short-term support for the isolated or combined failure of the heart or lungs	Poor survival Infection Bleeding Renal failure Neurologic sequelae
VAD	Ventricle unloading Cardiac output augmentation Adverse event minimization Lower mortality Life quality improvement	Pump thrombosis Gastrointestinal bleeding Infection Stroke
Heart transplantation	Ultimate therapeutic approach	Increased risks of bleeding

TE, thromboembolic events; ECMO, extracorporeal membrane oxygenator; VAD, ventricle-assist devices.

### Antithrombotic medications

The management of Fontan-associated thrombosis typically includes anticoagulant therapy, thrombolytic treatment, and embolectomy. The decision-making is heavily determined by the potential pathogenic factor and the size and location of the thrombus. Vitamin K antagonists are recommended with Class I in those with prior atrial arrhythmias, TE, and suspected or diagnosed thrombus according to the 2018 ACHD guidelines. As for those with asymptomatic thrombus, antithrombotic treatment is recommended with Class IIb (6).

As for those without TE yet, thromboprophylaxis may be paramount and appropriate in Fontan management, given the significant association of TE with morbidity and mortality. Indeed, prophylactic administration of antithrombotic drugs has increasingly become routine in Fontan patients as it was associated with a lower risk of hospitalization and death, and a lower TE rate (37). Aspirin and warfarin are frequently used in this context; however, which thromboprophylaxis agent is a more appropriate option remains under investigation. More and more evidence indicated that antithrombotic efficacy regarding the prevention of early or late TE was not significantly different between aspirin and warfarin (38). Of note, warfarin-based thromboprophylaxis perhaps increases risks of bleeding and reduction of bone mineral density in contrast to aspirin-based thromboprophylaxis. And cerebrovascular Injuries such as white matter injury and microhemorrhage represent the major issue in Fontan patients regardless of prophylactic administration of drugs.

Based on current evidence, several guidelines have been proposed to guide the decision-making in the thromboprophylaxis management of Fontan patients. UFH or ASA combined with an additional vitamin K antagonist are recommended for those Fontan children in the Guidelines from the American College of Chest Physicians in 2012 (39). A Scientific Statement from the American Heart Association in 2013 recommends LMWH or warfarin in the initial 3–12 months after Fontan surgery, and long-term thromboprophylaxis using antiplatelet medications, or long-term thromboprophylaxis using warfarin for those with TE risk factors. Administration of aspirin for thromboprophylaxis with a dose of 1–5 mg/kg and a maximum of 150 mg every day is recommended in Fontan patients. If selecting warfarin, it would be appropriate to target the INR between 2 and 3 by an initial loading dose of 0.1 mg/kg every day and a subsequent personalized maintenance dose. In addition, INR should be monitored daily until reaching a therapeutic target (40).

### Ablation

Catheter ablation has been attempted in eliminating arrhythmic burden in Fontan patients due to less discomfort and pain, lower mortality rate, and a shorter length of hospital stay. It has been demonstrated that catheter ablation is capable of reducing arrhythmia burden in AP Fontan, as evident by sustained reduction of arrhythmia scores at 3 months, 6 months, 1 year, and 2 years (41). Additionally, prophylactic ablation before Fontan surgery in selected patients is also anticipated. Indeed, recently Takeuchi et al. performed catheter ablation in FSV patients at high risk for supraventricular tachycardia prior to Fontan surgery, showing that pre-operative ablation significantly reduced the perioperative or post-operative occurrence of arrhythmia (42). The targeted arrhythmogenic area can be obtained through a retrograde aortic approach to the subaortic ventricle chamber crossing the aortic valve or an anterograde approach to pulmonary venous atrium *via* a fenestration, *trans-*baffle, or *trans-*conduit puncture into the atrium. When sufficient cavo-atrial overlap presents in ECC Fontan, a *trans-*caval puncture through a hepatic vein or inferior vena cava adjacent to the pulmonary venous atrium wall appeared to be feasible (43). On occasion, in the context of venous obstruction unable to obtain percutaneous vascular access, the transthoracic percutaneous route may be an alternative in successful mapping and ablation as of offering a rapid and direct approach to the pulmonary venous atrium.

Complications resulting from the ablation like complete atrioventricular block and residual inducible arrhythmias are major concerns. Despite the afore-mentioned attempts, the evidence regarding the efficacy and safety of intervention ablation is extremely limited.

### Pacing

Pacemaker implantation in patients with congenital cardiac malformation and arrhythmia benefits hemodynamic improvement, antiarrhythmic medication effect, and symptom relief. In Fontan patients, cardiac pacing remains challenging due to limited venous access for endocardial lead placement, as well as the necessity of sternotomy or thoracotomy for epicardial lead placement. Despite this, the implantation of anti-tachycardia pacing has been commonly performed during Fontan conversion to prevent and terminate future atrial tachyarrhythmias. The most common indication for cardiac pacing in Fontan patients is sinus node dysfunction. The improved hemodynamics resulting from pacing has been verified to benefit the management of PB and PLE.

To date, pacing lead implantation can be obtained by surgery or interventional catheterization through epicardial, transmural, and endocardial approaches. A higher reintervention rate of pacemakers was observed in epicardial lead implantation in contrast to endocardial lead implantation (44). There was also an increase in energy threshold and temporal reduction in impedance in epicardial leads compared with endocardial leads (45). Despite the longer longevity of pacemaker leads, transvenous endocardial lead implantation is technically challenging and increases the clot burden so that requiring additional anticoagulation. Herein, the knowledge gap regarding the ideal pacing method like epicardial or endocardial lead implantation still exists.

The outcome of permanent ventricular pacing may be disappointing as a consequence of an increase in the risk of transplantation and late death (46). To attenuate the negative impact caused by ventricular pacing, the apical was recommended as the optimal epicardial pacing site that is capable of avoiding ventricular stress augmentation (47).

### Mechanical circulatory support

Extracorporeal membrane oxygenator (ECMO) is a well-established modality with short-term support for the isolated or combined failure of the heart or lungs. Common indications of initiation of ECMO in post-operative CHD patients comprise extracorporeal cardiopulmonary resuscitation, hemodynamically unstable arrhythmias, refractory low cardiac output, thrombogenesis within the systemic-to-pulmonary shunts in FSV patients, persistent hypoxemia, and weaning failure from the cardiopulmonary bypass. ECMO support in FSV patients with Glenn or Fontan physiology is challenging owing to additional complexity in surgical anatomy and physiology. Technically, sufficient venous drainage through multi-site cannulation and venous decompression followed by appropriate ECMO management are imperative components in achieving successful ECMO. Veno-arterial ECMO (VA ECMO) is the common supportive modality, with a mortality rate of 57% (48). The poor survival and significant mortality were considered to be attributed to the increased possibility of ventricular distension, related sub-ideal coronary perfusion, and an imbalance between pulmonary and systemic blood flow (49). A longer time of cardiopulmonary bypass and birth weight <2.5 kg were identified to be independently related to the requirement of VA ECMO support after Norwood surgery (50). Of note, infection, bleeding, renal failure, and neurologic sequelae in terms of white matter injuries, seizures, strokes, and intracranial hemorrhages are well-recognized issues in VA ECMO support.

In contrast, venovenous ECMO (VV ECMO) is another supportive modality for FSV patients with pure ventilation or oxygenation impairment. Aydin et al. described the outcome of FSV patients with the respiratory disease under VV ECMO support, using the data from the Extracorporeal Life Support Organization Registry. It showed a survival rate of 48%, comparable to those FSV patients with cardiac failure undergoing VA ECMO (51). The renal injury and higher mean airway pressures were demonstrated to be associated with mortality, highlighting the future direction for survival improvement by minimizing the ventilator-induced damage. More investigations to enhance the management of such a population are required to define the cannulation strategy in terms of timing, type, and location.

The application of ventricle-assist devices (VAD) as a bridge to transplantation, recovery, or destination therapy has facilitated the management of advanced ventricular failure by minimizing adverse events, decreasing mortality, and improving life quality. In Fontan patients, it is also capable of improving the hemodynamics by unloading the FSV and augmenting the cardiac output. A recent retrospective report from the Advanced Cardiac Therapies Improving Outcomes Network registry showed that over 2/3 of recipients implanted with VAD could be successfully supported to undergo consequent heart transplantation (52). Technically, the implantation of VAD in Fontan individuals for subpulmonic circulation requires takedown of Hemi-Fontan or Fontan shunts, repair of the pulmonary artery, and reconstruction of the atrium. Device-related issues such as pump thrombosis, gastrointestinal bleeding, device-associated infection, and stroke exist. A stroke occurs more commonly in recipients with the centrifugal flow, while gastrointestinal bleeding appears to be frequent in recipients with the axial flow.

The type of implanted device is supposed to be fully considered because anatomy, size, and symptoms may vary and therefore complicate the device selection. Although it is in infancy, increasing case reports and series have demonstrated the successful support of several current available VADs in Fontan patients with ventricular failure, including the Berlin Heart EXCOR, HeartWare, and HeartMate 3. Current experience regarding the VAD support in Fontan patients is limited, and further investigation to decide the type of device, patient selection, and optimal timing is necessary.

### Heart transplantation

Heart transplantation represents an ultimate therapeutic approach to replace the originated heart in Fontan failure. Fontan patients are the most common and rapidly growing population in CHD referred for heart transplantation owing to inherent vulnerabilities, with a prevalence of 0.7–6.8% (53). Early investigation on post-transplantation outcomes in Fontan patients revealed a significantly reduced survival rate in contrast to other patients after heart transplantation (54). This could be perhaps attributed to under-identified liver failure, surgical complexity, and delayed referral (55). However, the overall survival rate seemed comparable between Fontan heart transplantation recipients and other CHDs in the later era, with the improvement and optimization in patient selection, pre-operative evaluation, and pre-operative and post-operative management (56). Precisely, it was reported that survival after heart transplantation in Fontan patients was 91, 78, and 71% at 1, 5, and 10 years, respectively (53). Requirement of MCS before the surgery, end-stage liver disease, older recipient age, AVVR greater than moderate, ventricular EF less than 20%, the interval between Fontan surgery and heart transplantation less than 10 years, and age at heart transplantation less than 18 were risk factors associated with post-operative mortality (57, 58). The identification of risk factors and even risk stratification can predict outcomes after transplantation, thereby allowing rational allocation of limited donor resources.

## Outlook

### Computational flow dynamics and 4-dimensional flow magnetic resonance in Fontan circulation

As mentioned above, the unique and unphysiological Fontan hemodynamics is the main trigger of a series of Fontan-associated complications. The characterization of abnormal hemodynamic or blood flow patterns is therefore vital. Computational flow dynamics (CFD) and 4-dimensional flow magnetic resonance imaging (4D flow MRI) have emerged as unprecedented imaging modalities, allowing the visualization and quantification of blood flow characteristics. 4D flow MRI refers to the acquisition of 3-dimensional time-resolved volume with velocity-encoding in all three directions throughout the cardiac cycle, while CFD involves the creation of a 3-dimensional model by numerical analysis based on 3-dimensional rotational angiography, CT, or MRI. Both CFD and 4D flow MRI have been increasingly applied in Fontan patients and shown abnormal blood flow patterns distinct from other populations, providing novel insights into the hemodynamic response and mechanism of Fontan failure. Reduced direct flow and augmented residual volume were principal hemodynamic features in Fontan patients with hypoplastic right heart syndrome and HLHS (59). There were also significantly higher ventricular kinetic energy and lower cardiac efficiency compared with healthy controls. As for those with RV-dominant morphology, higher peak diastolic kinetic energy was also detected (60). Fontan circulation was also inherent with increased intra-ventricular energy loss and disproportionate intra-ventricular energy loss relative to kinetic energy (61).

Apart from the elucidation of hemodynamic alteration in Fontan circulation, CFD and 4D flow MRI, capable of patient-specific computational modeling, can also aid in customized surgical planning for Fontan patients. The current optimization of patient-specific TCPC design focus on two major aspects: (1) minimization of the energy loss and (2) balance of hepatic blood distribution. In this regard, Trusty et al. modified the TCPC with bifurcated Y-grafts that directed the hepatic blood flow into both the right pulmonary artery and left pulmonary artery and explored computational fluid dynamics for the evaluation of energy loss and hepatic flow distribution. The results showed that a more balanced hepatic flow distribution was obtained without increasing TCPC resistance, highlighting the Y-graft modification value in Fontan completion (62).

Therefore, the application of CFD and 4D flow MRI will not only improve the understanding of hemodynamic mechanisms that predict the complications early but also benefit the design of patient-specific surgery plans. Ongoing methodological improvements would increase the reliability of hemodynamic characterization, the accuracy of the customized computational models, and the feasibility of the next-step surgical plan.

### Fontan ventricular assist device

Fontan ventricular assist device has emerged as an area of growing interest on account of its potential to decongest venous pressure, overcome the elevated pulmonary resistance, augment the cardiac output, and thereby address the Fontan circulatory insufficiency. It was initially described by de Leval to deal with the Fontan paradox in terms of coexisting reduced cardiac output and elevated central venous pressure in contrast to the conventional MCS support (63). Since then, the assist system has been further exploited, followed by a series of attempted innovations of the cavopulmonary assist pumps like micro-axial pumps and viscous Impeller pumps. Indeed, these pumps have been shown as a feasible and promising option for temporary support in failing Fontan patients. Of note, external power is required to drive the assist devices, which is associated with device-related infections. To eliminate the use of external power, Pekkan et al. designed an integrated aortic-turbine venous-assist device system that extracted a fraction of aortic blood to drive the aortic turbine. Consequently, this system demonstrated great hemodynamic performance as generating venous recovery with up to around 5 mmHg and meanwhile supplying complete caval blood flow (64). The long-term and durable cavopulmonary support by maintaining biventricular equivalency remains in infancy. It was shown that pressure step-ups at approximately 6 mmHg were appropriate to allow long-term biventricular maintenance under normal physiologic situations (65). However, the Fontan patients are heterogeneous subjects, varying in age, health status, and individual physical activity pursuits. Escher et al. manufactured a novel cavopulmonary assist system composed of a drive unit, electric motors, ceramic bearings, and hemocompatible titanium components. It allowed long-term cavopulmonary support at lower power consumption, accessible to a relative range of hemodynamic and physiologic situations in Fontan patients (66). Further development and refinement of pump devices to alleviate related complications, as well as to provide long-term support will enhance the translation of therapeutic right-side Fontan ventricular assist devices in failing Fontan circulation.

### Tissue-engineered vascular grafts

The EC Fontan involves the introduction of man-made grafts, homografts, or xenografts, which is disadvantageous due to the inability to grow, as well as susceptibility to develop TE, stenosis, chronic infection, and calcification. Tissue-engineered vascular grafts (TEVG) through the integration of synthetic or natural degradable scaffold and seeded cells hold considerable promise to address these obstacles. After implantation, the TEVG goes through the degradation and remodeling resulting from the induced host cell infiltration over time, ultimately replaced by the autologously regenerative vascular tissue. The neotissue formation was mediated by an early inflammation-driven response followed by a mechano-induced response (67). These grafts have shown moderate safety and feasibility in the clinical trial (68). Despite the encouraging outcomes, graft stenosis is a frequent complication that may compromise the long-term efficacy of TEVG. The TEVG stenosis formation was driven by the classic activation of host monocytes through TGF-β signaling pathways (69). Some strategies have been adopted to mitigate the development of graft stenosis and to improve graft performance, including the administration of cilostazol, optimization of seeding cell number and type, and scaffold refinement by computational modeling. Another recent progress is the generation of a contractile Fontan conduit that can serve as an assist device to aid in the pulmonary circulation. Park et al. proposed a versatile molecular design strategy to produce a pulsatile Fontan conduit by a generation of engineered heart tissue composed of porcine extracellular matrix, primary cardiac fibroblasts, and human-induced pluripotent stem cell-derived cardiomyocytes wrapped with decellularized human umbilical artery. The pulsatile conduit not only addressed the limitations of previous attempts but also exhibited effective mechanical and electrical function, which may be a solid foundation of therapeutic tissue-engineered pulsatile conduit in FSV patients (70). Future explorations on mitigation of the graft stenosis and generation of a more ideal contractile conduit to improve pressure and flow in pulmonary circulation will broaden the TEVG clinical opportunities.

### Stem cell therapy

Stem cell therapy holds promising potential, which has been employed to discover the underlying mechanism of malformation development, as well as preserve or improve ventricular performance in FSV patients, most commonly in HLHS. It is the most complex and common type of FSV abnormalities, characterized by underdevelopment of the mitral valve, LV, outflow structures, and aorta. Despite advancements in medical management and surgery evolution that increase the likelihood of surviving into adulthood, the RV in the HLHS physiology inherently presents vulnerability and intolerance to chronic pressure overloading, therefore predisposed to early ventricular failure. There was a reduced angiogenic response of RV in the HLHS patients in the context of pressure overloading, thereby impeding the delivery of nutrients and oxygen to cardiomyocytes and further leading to myocardial dysfunction (71). The development of RV was also attributed to the antioxidant response reduction, change from oxidative mitochondrial metabolism into less valid glycolytic metabolism, cardiomyocyte apoptosis, and myocardial fibrosis (72, 73). To date, diverse stem cells combined with or without biomimetic scaffold have been attempted to preserve or enhance the RV function *in vitro* and *in vivo*, mainly targeting the enhancement of angiogenesis and minimization of oxidative stress. Strikingly, some clinical trials have been sponsored and completed to boost the clinical translation of stem cell therapy in HLHS treatment (74).

Although tremendous technical obstacles, as well as safety and efficacy, remain in the current era, stem cell therapies and cardiac regenerative medicine are believed to benefit the future management and treatment of HLHS. The clinical translation of stem cell therapy in HLHS treatment can be expected if issues such as the timing of intervention, method, dosing of administration, and optimal stem cell type are defined in the future.

## Author contributions

HY was responsible for the conception and design of the present work. JM performed the literature search and drafted the manuscript. All authors took part in manuscript revision and approved the final version of the manuscript.
